# T405, a New Penem, Exhibits *In Vivo* Efficacy against M. abscessus and Synergy with β-Lactams Imipenem and Cefditoren

**DOI:** 10.1128/aac.00536-22

**Published:** 2022-05-31

**Authors:** Binayak Rimal, Hunter R. Batchelder, Elizabeth Story-Roller, Chandra M. Panthi, Chavis Tabor, Eric L. Nuermberger, Craig A. Townsend, Gyanu Lamichhane

**Affiliations:** a Division of Infectious Diseases, grid.471401.7grid.21107.35grid.471401.7grid.21107.35 Johns Hopkins University School of Medicine, Baltimore, Maryland, USA; b Department of Chemistry, Johns Hopkins University grid.471401.7grid.21107.35grid.471401.7grid.21107.35 , Baltimore, Maryland, USA

**Keywords:** β-lactam, penem, T405, efficacy, dual β-lactam, *Mycobacterium abscessus*, *Mycobacteroides abscessus*

## Abstract

Mycobacteroides abscessus (*Mab*) is an emerging environmental microbe that causes chronic lung disease in patients with compromised lung function such as cystic fibrosis and bronchiectasis. It is intrinsically resistant to most antibiotics, therefore there are only few antibiotics that can be repurposed to treat *Mab* disease. Although current recommendations require daily intake of multiple antibiotics for more than a year, cure rate is low and often associated with significant adverse events. Here, we describe *in vivo* efficacy of T405, a recently discovered β-lactam antibiotic of the penem subclass, in a mouse model of pulmonary *Mab* infection. Imipenem, one of the standard-of-care drugs to treat *Mab* disease, and also a β-lactam antibiotic from a chemical class similar to T405, was included as a comparator. Probenecid was included with both T405 and imipenem to reduce the rate of their renal clearance. T405 exhibited bactericidal activity against *Mab* from the onset of treatment and reduced *Mab* lung burden at a rate similar to that exhibited by imipenem. The MIC of T405 against *Mab* was unaltered after 4 weeks of exposure to T405 in the lungs of mice. Using an *in vitro* assay, we also demonstrate that T405 in combination with imipenem, cefditoren or avibactam exhibits synergism against *Mab*. Additionally, we describe a scheme for synthesis and purification of T405 on an industrial scale. These attributes make T405 a promising candidate for further preclinical assessment to treat *Mab* disease.

## INTRODUCTION

Mycobacteroides abscessus (*Mab*, also known as Mycobacterium abscessus) is an emerging opportunistic pathogen whose incidence has been rising (1). It causes a wide range of chronic infections whose clinical presentations can resemble disease resulting from infection by Mycobacterium tuberculosis and nontuberculous mycobacteria such as Mycobacterium avium (2–4). Individuals with structural lung diseases such as bronchiectasis, cystic fibrosis and chronic obstructive pulmonary disease are at higher risk of developing lung disease from *Mab* infection (5). In these settings, *Mab* infections result in rapid decline in lung function and are difficult to cure with existing treatment regimens (2–4). Although infections of the lungs comprise the majority of *Mab* infections that require treatment, infections of the joints and soft tissue resulting from surgical procedures have been described (6–9). Recent accounts have also reported clusters of outbreaks, including in health care settings (10). Often infections are chronic and associated with unacceptably high rates of morbidity and mortality.

One of the primary reasons for the high morbidity and mortality rate is the lack of effective treatment. There are no FDA-approved antibiotics based on clinical trials to treat *Mab* infection. The existing treatments are comprised of repurposed antibiotics and recommended based on expert opinions. Since *Mab* is intrinsically resistant to most available antibiotics, only a limited set can be called upon to treat this disease. The treatments available to *Mab* patients are sub-optimal for the following reasons: (a) need for prolonged treatment periods, (b) frequent adverse side effects, (c) complicated logistics of long-term outpatient intravenous drug treatment, and (d) inability to produce a stable cure (11–15). These treatments are associated with cure rates as low as 30–50% (16) and sputum conversion rates as low as 25% (17). For these reasons, *Mab* has been declared ‘an antibiotic nightmare’ (5) and ‘an environmental bacterium turned clinical nightmare’ (18).

Historically, owing to the presence of native β-lactamases, β-lactams are seldom considered for treatment of mycobacterial infections (19–21). In *Mab*, the chromosomally encoded β-lactamase, Bla_Mab_, exhibits strong inactivating activity against a broad range of β-lactams (22). However, among the repurposed antibiotics to treat *Mab* disease, several belong to the β-lactam class and exhibit high efficacy. These include imipenem and cefoxitin, which are included in the ATS/IDSA treatment guidelines for *Mab* disease (11, 23, 24). Other frequently considered β-lactams include doripenem, ceftazidime-avibactam, imipenem-relebactam and meropenem-vaborbactam. In addition to imipenem, which is already included in current treatment recommendations, other carbapenems such as biapenem and tebipenem-avibactam also exhibit low MICs against *Mab* (25, 26). Additionally, several combinations of dual β-lactams with or without a β-lactamase inhibitor exhibit synergy against a broad spectrum of *Mab* isolates both *in vitro* and *in vivo* (27–32). This emerging evidence has rekindled interest in the potential of β-lactams for treating *Mab* diseases.

T405 is a newly discovered β-lactam of the penem subclass (33). Its MIC against a collection of *Mab* clinical isolates, including those classified as drug-resistant is 1 to 8 μg/mL. The MIC_90_ of T405 against *Mab*, 8 μg/mL, is significantly lower than the MIC_90_ of imipenem and cefoxitin, 32 μg/mL, and 64 μg/mL, respectively, indicating a more potent *in vitro* activity against *Mab* than these two β-lactams included in the current recommendation. Moreover, T405 is highly resistant to Bla_Mab_ and exhibits favorable pharmacokinetics as well as toxicity profile at elevated dosage in mice, making it an excellent candidate for further preclinical evaluation (33).

In this study, we assessed the efficacy of T405 against *Mab* in a mouse model of pulmonary *Mab* infection. Larger scale preparation of T405 was adapted from the previous synthesis (33) to be more industrially applicable to allow for larger quantities to be produced in a formulation that is suitable for an *in vivo* model. We also determined if exposure to T405 in the mouse lungs alters the MIC of T405 against *Mab*. As T405 exhibits *in vitro* activity against *Mab*, we also assessed if a combination of T405 and another β-lactam exhibits synergy against *Mab.*

## RESULTS

### Large scale synthesis of T405.

**FIG 1:**
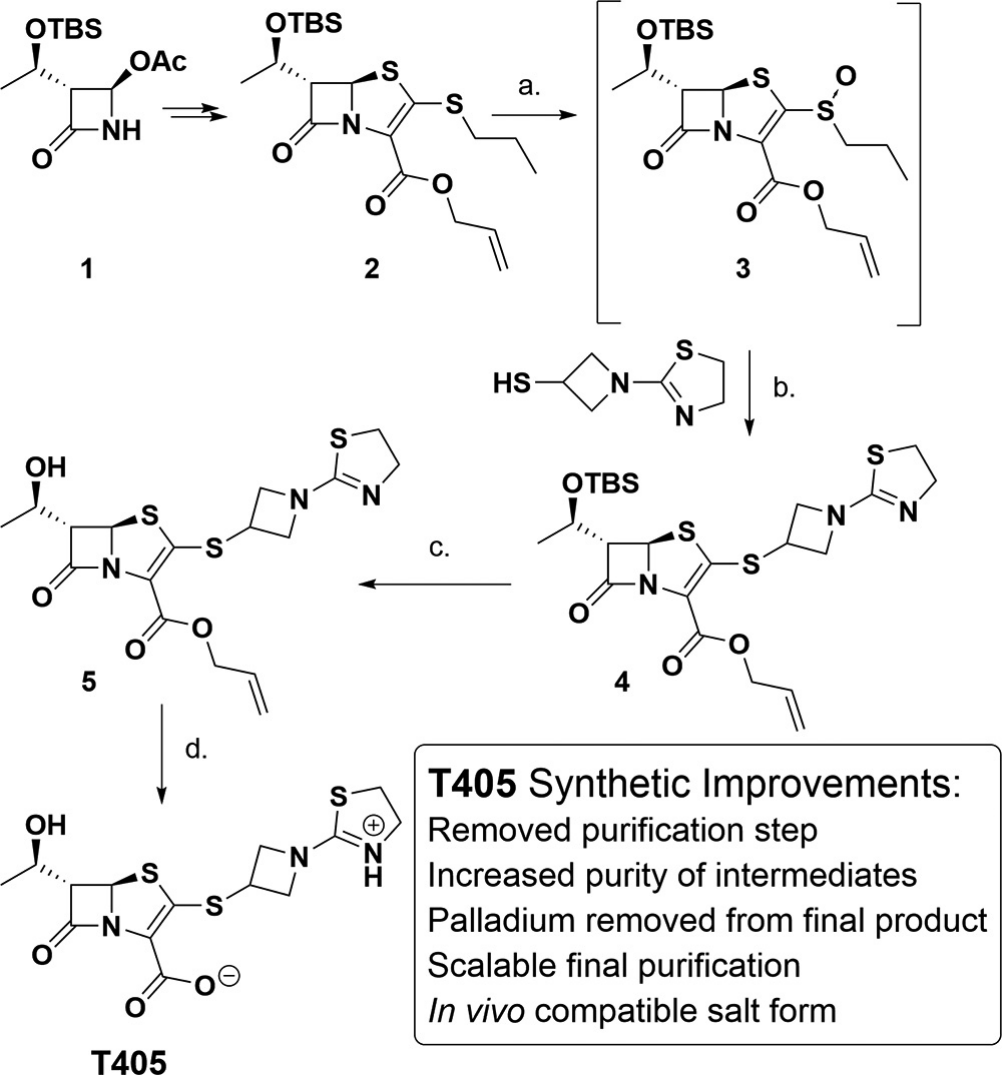
Large-scale synthesis of T405. (a) Urea-H_2_O_2_, HFIPA, (b) DIPEA, CH_3_CN, 66% yield over 2 steps, (c) TBAF, AcOH, THF, 80% yield, (d) CH_2_Cl_2_, H_2_O, Pd(PPh_3_)_4_, benzene sulfenic acid, 58% yield.

The synthesis of T405 was redesigned to allow for its multigram scale production (Fig 1). The new synthesis of T405 deviates at the common intermediate 2 where the silyl protecting group is left intact on the alcohol while the oxidation of the side chain sulfur is carried forward. This change results in the transient intermediate 3 which was found unnecessary to isolate before proceeding to the next reaction. Consequently, the β-addition-elimination sidechain swap reactions could proceed on the crude oil to form 4 in one overall step from 2. In addition to allowing for the removal of a lengthy and costly silica-gel flash chromatography step, the silyl ether 4 allowed the molecule to be triturated in higher purity. A similar strategy was developed in the industrial synthesis of sulopenem (34). With compound 4 in hand, the silyl ether is removed to yield compound 5, the common intermediate of the previously described synthesis.

When preparing drugs for animal efficacy studies the compound formulation must be rid of heavy metal ion impurities and be in an appropriate salt form for the *in vivo* study. To accommodate these restrictions the palladium-catalyzed deallylation and subsequent purification of T405 was redesigned. The new synthesis utilizes a biphasic, palladium-catalyzed deallylation designed to keep palladium in the organic layer while the product is partitioned into the aqueous layer. Moreover, the resulting product is incubated with the palladium-scavenging macroporous polystyrene-bound trimercaptotriazine (MP-TMT) resin to remove any residual heavy metal ions. A similar approach was reported for the industrial synthesis of faropenem (35). Lastly, the compound was purified on multigram scale using a reusable HP-20 resin and a gradient elution of deionized water to ethanol yielding the zwitter ionic T405. This process differed from the high-performance liquid chromatography that was used in the original synthesis, which faced a scaling limitation and yielded product containing the toxic trifluoroacetate counter ion. Detailed information on synthetic steps, intermediates and their characterizations are included in Supplementary Information.

### Efficacy of T405 versus *Mab* lung infection in C3HeB/FeJ mice.

Efficacy of T405 was assessed against the M. abscessus reference strain ATCC 19977 in a mouse model of pulmonary infection (36) using a protocol identical to that used to evaluate the efficacy of β-lactams from the carbapenem class against *Mab* (28). Mice were infected with ATCC 19977 *via* an aerosol route and randomized into three groups. Mice in the negative-control group received 1× phosphate-buffered saline (PBS), which was used as solvent for imipenem and T405. Imipenem, an antibiotic that is used in standard-of-care regimens to treat *Mab* disease and also a β-lactam (24), was administered to mice in the positive-control comparator group. The mice in the test group received T405. Based on a prior pharmacokinetics study of T405 in mice, imipenem and T405 were administered with probenecid, an agent that inhibits renal clearance of β-lactams (33). PBS, imipenem and T405 were administered via subcutaneous injection into the rear dorsal flank, and probenecid was administered via oral gavage. Mode and frequency of treatment administration and handling of mice were identical among the three groups to ensure that treatment type was the only variable.

**FIG 2:**
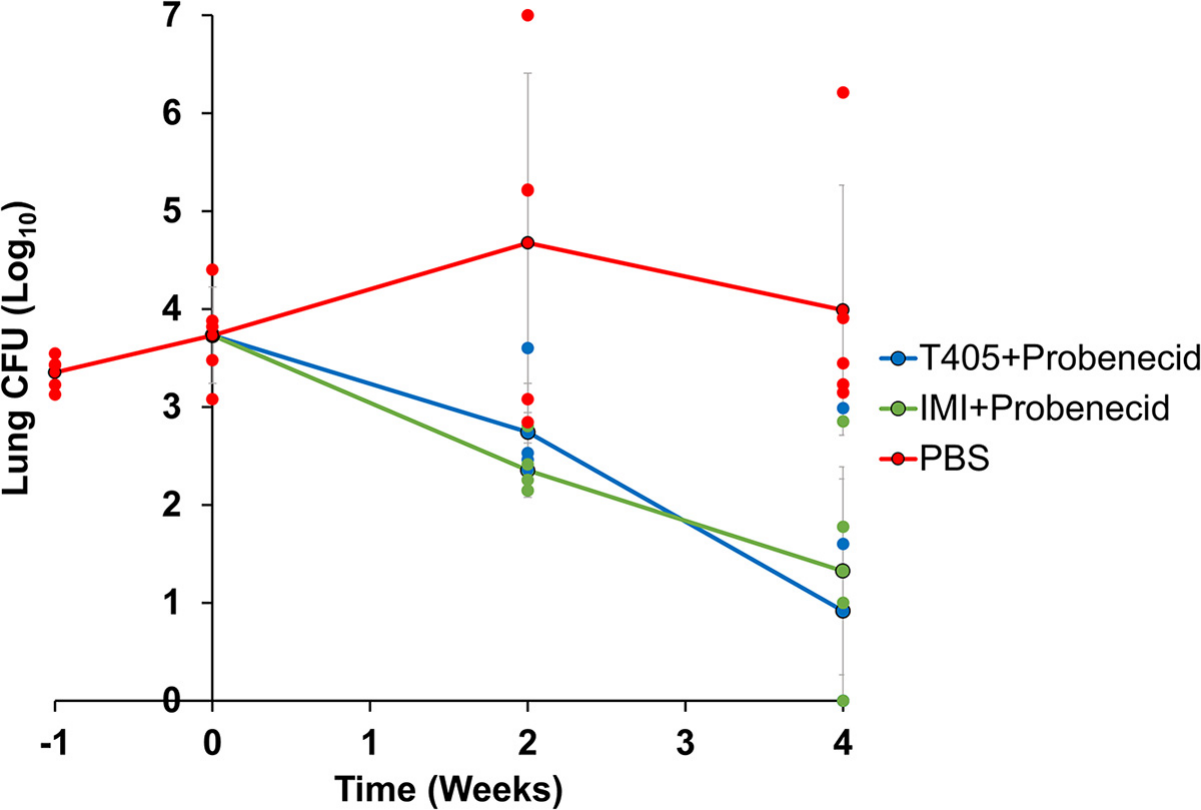
M. abscessus burden in the lungs of C3HeB/FeJ mice treated with T405+probenecid or imipenem+probenecid or phosphate-buffered saline. Week −1 represents 1 day following infection. Week 0 represents the day treatments were initiated. Weeks +2 and +4 represent 2 and 4 weeks following treatment initiation. Data shown was derived from five mice per treatment group per time point. Each dot represents *Mab* CFU in the lungs of a mouse. Each line represents mean *Mab* CFU in the lungs of the corresponding treatment group. Vertical bars represent standard deviation of the mean CFU at each time point. PBS, phosphate-buffered saline (negative-control comparator); IMI, imipenem (positive-control comparator).

As expected from prior studies (28) in mice treated with PBS, *Mab* lung burden gradually increased through the first 3 weeks following infection and thereafter decreased slightly (Fig. 2). Overall, *Mab* burden was maintained in the lungs of mice during the course of study and did not alter significantly as would be expected during a chronic *Mab* infection. In mice treated with imipenem+probenecid, the positive-control comparator group, *Mab* lung burden gradually declined over the course of the experiment and reproduced prior observations of imipenem against *Mab* ATCC 19977 (28, 37). Similar to the effect of imipenem, T405 also exhibited bactericidal activity throughout the treatment duration as *Mab* lung burden in mice receiving T405+probenecid gradually declined following the onset of treatment. In comparison to the negative-control group, 2.66 log_10_ and 3.07 log_10_ reduction in lung *Mab* CFU were associated with imipenem and T405 treatment, respectively. Although the reductions in lung *Mab* burden in imipenem and T405 treated groups were statistically not distinct, the mean *Mab* burden in mice treated with T405 was 0.42 log_10_ lower than in mice treated with imipenem demonstrating that efficacy of T405+probenecid was comparable or superior to the standard-of-care, imipenem, combined with probenecid. The *Mab* burden in the lungs of mice treated with T405+probenecid was significantly lower than PBS-treated negative controls throughout the treatment period (*P*-value 0.0061, PBS *versus* T405, week four time point) thereby demonstrating efficacy associated with T405 against *Mab*.

Lung pathology such as granulomas, bronchiectasis, brochiolectasis, and bronchiolitis are the most frequently observed hallmarks in humans with *Mab* lung disease (38) and were, therefore, considered in our study as pathological endpoints. Two mice per treatment group at the final time point were allocated for lung histopathological assessments. Areas of cellular infiltration and consolidation resembling a granuloma were observable in the lungs of mice treated with T405 and imipenem, but they were more diffuse and disorganized, and clear alveolar spaces were more numerous compared to the lungs of mice treated with PBS in which a more defined granuloma with intense cellular infiltration was visible (Fig. S1). *Mab* bacilli were readily observable in the lungs of mice that received PBS (Fig. S2). The majority of *Mab* bacilli appeared to be intracellular but *Mab* that were not associated with lung cells were also not uncommon. We were unable to observe *Mab* bacilli in the lung sections of mice treated with T405 or imipenem.

### MIC of T405 versus *Mab* is not altered following 4-week daily exposure in mouse lungs.

Although T405 exhibited a bactericidal activity and gradually reduced lung burden of *Mab*, ~1 log_10_ surviving CFU were recovered at the end of the treatment duration. We investigated whether the surviving *Mab* CFU were resistant to T405 and therefore would exhibit significantly higher T405 MIC. We randomly selected a total of eight *Mab* colonies recovered from the lungs of all five mice sacrificed at the final time point in the study. This time point was selected as it represents the longest duration of exposure of T405 to *Mab. Mab* exhibits heterogeneous colony morphology with smooth and rough surfaces. The distribution of smooth versus rough colonies of the input culture of *Mab* that was used to infect mice was 84% versus 16%, respectively. We selected six colonies with smooth appearance and two with rough appearance to represent the distribution of the smooth versus rough colonies in recovered CFU. We determined MICs of T405 for these eight isolates and for the parent isolate simultaneously (Table S1). The MICs of T405 against the eight isolates were in the range of 0.5 to 1.0 μg/mL and hence indistinguishable from the MIC of T405 for the parent isolate. Therefore, exposure to T405 in the lungs of mice for 4 weeks did not alter its MIC against *Mab*.

### T405 exhibits synergy with imipenem and cefditoren.

We assessed if T405 exhibits synergy, indifference or antagonism when combined with β-lactam antibiotics. The MICs of T405 as well as nine cephalosporin, six carbapenem and one penem antibiotics against *Mab* reference strain ATCC 19977 were determined. Cephalosporins included in this assessment were cefadroxil, cefprozil, cefuroxime, cefixime, ceftibuten, cefdinir, cefditoren, cefpodoxime and cefoxitin. Carbapenems included were ertapenem, meropenem, imipenem, doripenem, biapenem and tebipenem. Faropenem, the only commercially available penem, was also included. A checkerboard assay (39) was subsequently used to assess *in vitro* activity of T405 in combination with these β-lactams, as well as the β-lactamase inhibitor avibactam, against the reference strain. The fractional inhibitory concentration index (FICI) was calculated for each combination of drugs that inhibited *Mab* growth at less than one-half the MIC of each individual drug. The FICI is a mathematical representation of the activity contributed by each drug in the combination. The most conservative interpretation of FICI was used, in which ≤0.5 indicates synergism, >0.5 to 4 indicates indifference, and >4 indicates antagonism (40).

Of the 16 combinations evaluated, three combinations exhibited synergy against ATCC 19977. These synergistic combinations included T405 plus imipenem, T405 plus cefditoren, and T405 plus avibactam (Table 1). However, the degree of the synergy differed between the paired combinations. The combinations of T405 and imipenem, as well as T405 and avibactam, exhibited greater synergy compared to T405 plus cefditoren. The FICIs of T405 in combination with cefadroxil or cefprozil or cefuroxime or cefixime or ceftibuten or cefdinir or cefpodoxime or cefoxitin or ertapenem or meropenem or doripenem or biapenem or tebipenem against *Mab* ATCC 19977 were >0.5 to 4, and therefore, the activities of these combinations were indifferent.

**TABLE 1:** Activity of T405 in combination with imipenem, cefditoren or avibactam^*a*^

Drug combination	Fractional inhibitory concentration index (FICI)			Result interpretation
	Replicate 1	Replicate 2	Avg FICI	
T405 + avibactam	0.266	0.266	0.266	Synergy
T405 + imipenem	0.281	0.266	0.274	Synergy
T405 + cefditoren	0.375	0.5	0.438	Synergy

a An average fractional inhibitory concentration index (FICI) was determined for each complex from two biological replicates. FICI of ≤0.5 was interpreted as synergy, >0.5 to 4 as indifference, and >4 as antagonism (40).

## DISCUSSION

β-lactam antibiotics exert their antibacterial activity by interfering with the biosynthesis and metabolism of bacterial cell wall peptidoglycan (41–43). The peptidoglycan of mycobacteria is atypical as the majority of the interpeptide linkages are between *meso*-diaminopimelic acid^3^ of one stem peptide and *meso*-diaminopimelic acid^3^ of an adjacent stem peptide generated by l,d-transpeptidases (44–46). This enzyme class is distinct from d,d-transpeptidases that generate interpeptide linkages between *meso*-diaminopimelic acid^3^ of one stem peptide and d-alanine^4^ of an adjacent stem peptide (47–49). Recent studies demonstrated that among β-lactams, carbapenems and penems are the most potent inhibitors of mycobacterial l,d-transpeptidases (29, 50–52). Among these, faropenem, a penem, inhibits *Mab* l,d-transpeptidases most effectively (52, 53). While faropenem exhibits favorable MICs against M. tuberculosis (25, 54), whether it has efficacy in treating tuberculosis is unclear (55, 56). We leveraged precedents on M. tuberculosis l,d-transpeptidases and carbapenems/penems guided by crystal structures (52, 53, 57–62) to develop improved inhibitors of M. tuberculosis l,d-transpeptidases with the hypothesis that unique penems may exhibit improved potency against this microbe. Similar attempts at developing unique and potent inhibitors of l,d-transpeptidases using the β-lactam core have been reported independently (63–65). While a select few new penems exhibited desirable MICs against M. tuberculosis, we were gratified to find that one of these penems, T405, had MICs lower than those of imipenem and faropenem against a collection of *Mab* isolates (33). Since imipenem is currently considered a first-line drug for *Mab* disease (24) and faropenem is the only marketed member of the penem subclass, T405 has potential as a penem with improved activity against mycobacteria and warrants further preclinical assessment.

Efficacy assessments comprise one of the first steps in the preclinical phase of drug development. To generate large amounts of T405 required for this assessment in an animal model of *Mab* disease, it was necessary to adapt the synthesis described in the initial report (33). The scheme described here is industrially applicable and permits synthesis and purification of T405 on large scale to enable *in vivo* testing. T405 is highly soluble in water and can be readily administered to animals. To match the dosing frequency of the positive-control drug imipenem (24), T405 was administered twice daily, 7 days a week. The 300 mg/kg dose of T405 used in this study was adapted from our prior pharmacokinetics assessment of T405 in mice to optimize time above MIC as this aspect is known to be an important driver of β-lactam efficacy (33). Twice daily subcutaneous administration of T405 dissolved in 1× PBS, pH 7.4, was well tolerated by mice during the entire 4-week treatment duration. There was no evidence of irritation at the site of T405 injection or other observable adverse effects. As probenecid is often included to reduce the rate of renal clearance of imipenem (66), we also included probenecid with imipenem and T405. Probenecid did not exhibit activity against *Mab* ATCC 19977 in a MIC determination assay (MIC > 512 μg/mL), therefore, it was not included with PBS in the efficacy study.

Microbiological and lung histopathological assessments were used to evaluate the efficacy of T405 against *Mab* lung infection. *Mab* burden in the lungs of mice represented the microbiological endpoint. T405 exhibited bactericidal activity throughout the 4-week treatment period. Although this period was insufficient to demonstrate if T405 could sterilize the lungs of mice, the results were sufficiently clear to demonstrate proof-of-concept of efficacy of T405. In PBS treated group, there was >3 log 10 difference between mice with the lowest and highest lung *Mab* burden. Similar observations have been reported before in C3HeB/FeJ mice, but the lung CFU burden was particularly variable for *Mab* strain ATCC 19977 (28, 37). Although cellular infiltration in the lung lesions of PBS-treated negative-control mice was more intense, we only observed one small necrotic granuloma in the lungs of mice representing this cohort despite harboring ~3 log_10_ higher *Mab* burden compared to T405- or imipenem-treated mice. In a prior study based on the identical protocol, the presence of a small granuloma at 3-week time point postinfection, and large organized histiocytic granulomas at 7-week time point were described (36). The presence of only one small granuloma in the PBS-treated mice is in agreement with the prior study. The lung parenchyma of mice treated with T405, and imipenem did not appear remarkably different from those of mice treated with PBS. There were areas of cellular infiltration and consolidation in the lungs of mice treated with T405 and imipenem. As a longer infection period was described to be necessary to observe large and distinct granulomas in the lungs of *Mab* infected C3HeB/FeJ mice (36) and resolution of established lung pathology typically lags behind changes in bacterial viability in response to treatment, the 4-week duration of our study with its primary focus on a microbiological endpoint was insufficient to differentiate tissue pathology between different treatment groups. As this is the first investigation to assess efficacy of T405 against *Mab*, we limited the scope of the study to proof-of-concept of T405 activity to treat *Mab* lung infection in a mouse model of the disease.

No acid-fast bacilli (AFB) were observed in the lung sections of mice treated with T405 and imipenem while there were numerous AFB, both intracellular and extracellular in the lungs of mice that received PBS only. The following are two likely explanations for this observation. It is most likely that the lung sections sampled did not include the region that harbored *Mab* as on average only ~1 log_10_ CFU were recovered from the lungs of mice in the T405 or imipenem treatment group. However, the possibility that *Mab* bacilli existed in the sampled lung sections but could not be stained with AFB stain cannot be ruled out. Imipenem and T405, as with other β-lactams, can be expected to act on the *Mab* cell wall and may have modified it sufficiently to alter incorporation of the AFB stain (54). Overall, both the microbiological and histopathological endpoints associated with T405 were not significantly different from those observed with the standard-of-care treatment imipenem.

Synergy between two β-lactams, also known as dual β-lactam synergy, has been demonstrated against *Mab* (27–31, 53) as well as other bacteria (67). These precedents were the basis for assessing the combined activity of T405 and select β-lactams from the cephalosporin, carbapenem and penem classes against *Mab.* Penicillins could not be included within the scope of the current study as the combinations would be numerous and beyond the exploration aimed for this study. Therefore, combined activity of T405 and penicillins against *Mab* can be evaluated in future studies. As T405 exhibited synergy with imipenem and cefditoren, these combinations may provide a pathway for further improving potency of regimens to treat *Mab* disease. However, additional studies will be required to determine if the combinations of T405 with imipenem or cefditoren act synergistically *in vivo* and against a wide range of *Mab* isolates.

We also assessed the combined activity of T405 with avibactam, an agent known to inhibit Bla_Mab_ (68). Synergy between avibactam and β-lactams cefuroxime, ertapenem, imipenem, biapenem and tebipenem against *Mab* has been reported (27, 69). In our recent study, we assessed the MICs of T405 alone and in combination with a fixed concentration of 4 μg/mL avibactam against a panel of 10 *Mab* clinical isolates (33). As avibactam reduced MIC of T405 by 4-fold against *Mab* strain ATCC 19977 but failed to significantly reduce MICs against most other clinical isolates, we determined the FICI of T405 and avibactam combination using a checkerboard assay. The FICI of 0.266 for T405 and avibactam against ATCC 19977 confirms synergy against this isolate. Although it is likely that synergism between T405 and avibactam is limited to a minority of *Mab* isolates, the MIC of T405 alone is sufficiently low and therefore may not require a companion β-lactamase inhibitor. Overall, the efficacy of T405 in comparison to imipenem and its synergism with imipenem and cefditoren make T405 a promising candidate for further preclinical development to treat *Mab* disease.

There were additional practical limitations to our study. Only a single dose and dosing frequency was assessed as >10 g of T405 would be necessary to consider two or more dosing regimens. Synthesis of such amounts is rate-limiting in an academic setting. *Mab* strains isolated from patients are known to exhibit heterogeneity in β-lactam susceptibility (70). Therefore, efficacy assessments against a more diverse set of isolates would have generated additional insight into the usefulness of T405 against a spectrum of *Mab* clinical isolates. However, as a proof-of-concept study, we limited the scope of the present study to assessing efficacy and combination activity against the *Mab* reference strain ATCC 19977. Similarly, we assessed *in vitro* activities of T405 in combination with representatives of β-lactam subclasses as, including more agents from this class was beyond the scope of the current study. Whether T405 exhibits synergy, indifference or antagonism with other antibiotics included in the current recommendations for treating *Mab* disease will also require additional studies. We posit, however, that findings from this study provide a strong premise for assessing T405 against additional *Mab* isolates in future studies.

## MATERIALS AND METHODS

### Preparation of T405.

T405 was synthesized, purified, and authenticated as described in the Supplementary Information.

### Bacterial strains and *in vitro* growth conditions.

M. abscessus strain ATCC 19977 was used as it has been historically considered a reference in *Mab* studies (71). This strain was procured from the ATCC (Manassas, VA) and authenticated by genome sequencing (30). Cation Adjusted Mueller-Hinton Broth (CAMHB) (catalog no. 90922, Sigma-Aldrich) or Middlebrook 7H9 broth (catalog no. 271310, Difco), as specified for an assay, was used to grow *Mab.* Middlebrook broth was supplemented with 0.5% glycerol, 10% albumin-dextrose-salt enrichment and 0.05% Tween 80 as described (72). *Mab* cultures were grown in an orbital shaker at 220 RPM, 37°C. Imipenem was procured from Octagon Chemicals Limited. Middlebrook 7H11 selective agar (catalog no. 283810, Difco), supplemented with 0.5% glycerol, 10% albumin-dextrose-salt enrichment, 0.05% Tween 80, 50 μg/mL carbenicillin (catalog no. C46000, Research Products International) and 50 μg/mL cycloheximide (catalog no. C7698, Sigma-Aldrich) was used to recover *Mab* from mouse lungs. All other β-lactam antibiotics were procured from Sigma-Aldrich or Octagon Chemicals. Probenecid was procured from Sigma-Aldrich (catalog no. P8761).

### MIC determination.

To determine the MIC of T405 against *Mab* isolates, the standard broth microdilution assay (73, 74) with parameters specified by the CLSI guidelines for *Mab* (75) was used. T405 was dissolved in sterile deionized water and passed through 0.22 μm cellulose filter (catalog no. 8160, Costar, Corning). Two-fold serial dilution of T405, from 64 to 0.06 μg/mL, was prepared in CAMHB, in 200 μL final volume in each well in a round-bottom 96-well culture dish (catalog no. 229190, CellTreat). Each *Mab* isolate grown in CAMHB broth to exponential phase was used to prepare a diluted suspension and 10^5^ *Mab* CFU was inoculated into each well. As negative and positive controls, two wells containing CAMHB broth alone and two wells containing CAMHB broth inoculated with 10^5^ CFU of corresponding *Mab* isolate, respectively, were included and incubated at 30°C, 72 h in accordance with CLSI guidelines. To determine growth of *Mab* or lack thereof, a Sensitive Manual Viewbox was used. The lowest concentration at which growth of *Mab* was not observed was recorded as the MIC of T405. MIC assay was undertaken simultaneously for all eight *Mab* isolates recovered from the lungs of mice and the parent isolate recovered at the beginning of the study. This assay was repeated three times. The data shown in Table S1 represent three biological replicates. MIC of probenecid against ATCC 19977 was determined using the identical protocol but 2-fold serial dilutions of probenecid ranging from 512 to 1 μg/mL was used.

### Checkerboard titration assay.

This assay was performed as described previously (27, 39). To determine the degree of synergy, two drugs were added to CAMHB broth in a 96-well plate, each starting at 2× MIC and serially diluted 2-fold down to 1/64× MIC; therefore, all possible 2-fold dilution combinations from 2× to 1/64× MIC were assayed. T405 was tested in combination with 16 β-lactam antibiotics (cefadroxil, cefprozil, cefuroxime, cefixime, ceftibuten, cefdinir, cefditoren, cefpodoxime, cefoxitin, ertapenem, meropenem, imipenem, doripenem, biapenem, faropenem, and tebipenem) and the β-lactamase inhibitor avibactam. *Mab* reference strain ATCC 19977 was grown in Middlebrook 7H9 broth to exponential phase as this broth permits planktonic growth of *Mab*, and therefore provides more accurate enumeration of CFU. This culture was used and diluted in CAMHB to 10^6^ CFU/mL and 10^5^ CFU was inoculated into each well, with positive and negative controls as described above. Samples were incubated at 30°C for 72 h and evaluated for *Mab* growth by visual inspection using a Sensititre Manual Viewbox. The fractional inhibitory concentration index (FICI) was calculated for each combination of drugs that inhibited *Mab* growth at less than one-half the MIC of each individual drug. FICIs of ≤0.5 were interpreted as synergy, FICIs of >0.5 to 4 as indifference, and FICIs >4 as antagonism, according to the most conservative interpretation recommended (40). All combinations with FICIs of ≤0.5 were tested in duplicate to confirm reproducibility, and an average FICI was calculated and reported here.

### Efficacy determination in mice.

C3HeB/FeJ mice, 5 to 6 weeks old, female (Jackson Laboratories, Bar Harbor, ME) were used as described in the protocol for a mouse model of pulmonary *Mab* infection (36) and in studies in which efficacies of β-lactams related to T405 were assessed or used as the positive-control comparator (28, 37). Beginning 1 week prior to infection and continuing throughout the study, 5 mg/kg/day dexamethasone (catalog no. D1756, Sigma-Aldrich) was dissolved in sterile 1× PBS, pH 7.4 (catalog no. 114-058-101CS, Quality Biologicals) was administered to each mouse, once daily, by subcutaneous injection in 0.1 mL volume into the hind dorsal flank using a 27-gauge needle (catalog no. 305620, Beckton and Dickinson). *Mab* strain ATCC 19977 was grown to exponential phase in Middlebrook 7H9 broth and was used to prepare a 10 mL infecting suspension at an optical density, A_600nm_ = 0.1. All mice in the study, *n* = 46, were infected simultaneously with an aerosolized suspension of *Mab* in an inhalation chamber according to manufacturer’s guidelines (Glas-Col, Terre Haute, IN). The infection cycle included preheating for 15 min, aerosol nebulization for 30 min, and cloud decay for 30 min, followed by surface decontamination for 15 min. Five mice were allocated for determination of *Mab* implantation following infection. Five additional mice were allocated for determination of *Mab* burden at 1 week following infection, the time at which antibiotic treatment was initiated. 1 week following infection, mice were randomly allocated into three groups of 12 mice per group. Treatment was administered twice daily, and 0.2 mL bolus of each treatment was administered in an identical way via subcutaneous injection in the rear dorsal flank. Mice in the negative-control group were administered 1× PBS, pH 7.4, as this buffer was used as the solvent for T405 and imipenem. Mice in the positive-control comparator group were administered 200 mg/kg imipenem + 250 mg/kg probenecid. Mice in the test group were administered 300 mg/kg T405 + 250 mg/kg probenecid. Based on a prior pharmacokinetics study of T405 (33), probenecid was included to inhibit renal clearance of T405 and imipenem. Probenecid suspension was prepared in 1× PBS, pH 7.4, and administered via oral gavage. *Mab* burden in the lungs of mice was determined at 24 h postinfection (designated week −1), 1 week following infection (week 0), and at two- and 4-weeks following treatment initiation (week +2 and +4, respectively). Five mice were sacrificed per time point per group. Lungs were homogenized in 1 mL final volume in 1× PBS, pH 7.4. Three 10-fold serial dilutions were prepared and inoculated onto Middlebrook 7H11 selective agar. CFU counts were recorded after incubation at 37°C for 5 d. CFU counts from each mouse lung were converted into CFU per lung, comprising the average of three consecutive steps of a 10-fold dilution series of a given lung sample. Mean CFU ± standard deviation in five mice per group per time point was plotted to determine *Mab* burden in the lungs of mice. Statistical analyses (*t*-tests) were undertaken with Microsoft Excel’s data analysis package for each time point for comparisons between treatment groups (Table S2).

### Ethics.

Animal procedures used in the studies described here were performed in adherence to the Johns Hopkins University Animal Care and Use Committee and to the national guidelines.

### Histopathological assessments.

Among the 12 mice in each treatment group described above, two mice per treatment group were allocated for histopathological analysis. These two mice, per group, were sacrificed at the end of the study period, week +4 time point, to assess the histology of lung tissue. Lungs were aseptically collected and incubated in 10% neutral buffered formalin solution (catalog number HT501128, Sigma-Aldrich) for 48 h as described (36). The fixed lungs were submitted to Johns Hopkins Tissue Histology Core for embedding in parafilm blocks. The Core sectioned and prepared standardized sections for unbiased sampling. These sections were stained with hematoxylin & eosin, Ziehl-Neelsen and Masson Trichrome and digital images of the sections were generated. Reviewers of the scanned images were blinded of the treatment group to remove any bias.
