# Supplementary material for: T405, a New Penem, Exhibits In Vivo Efficacy against M. abscessus and Synergy with β-Lactams Imipenem and Cefditoren
Source: Antimicrob Agents Chemother. 2022 May 31;66(6):e00536-22. doi: 10.1128/aac.00536-22 (PMC9211421; doi:10.1128/aac.00536-22)
Supplement: Supplemental file 1 — Tables S1 and S2, Fig. S1 and S2, and general methods and instrumentation. Download aac.00536-22-s0001.pdf, PDF file, 0.3 MB [file aac.00536-22-s0001.pdf]

SUPPLEMENTAL MATERIAL

T405, a new penem, exhibits efficacy against *M. abscessus* and synergy with β-lactams imipenem and cefditoren

Binayak Rimal, Hunter R. Batchelder, Elizabeth Story-Roller, Chandra M. Panthi, Chavis Tabor, Eric L. Nuermberger, Craig A. Townsend, Gyanu Lamichhane*

Table S1. MIC of T405 against *M. abscessus* isolates recovered from lungs of mice.

Mab isolate ID	MIC of T405 (µg/ml)			
	Replicate_1	Replicate_2	Replicate_3	Range
2201BR	0.5	0.5	0.5	0.5
2202BR	0.5	0.5	1.0	0.5-1.0
2203BR	1.0	0.5	1.0	0.5-1.0
2204BR	0.5	0.5	0.5	0.5
2205BR	0.5	0.5	1.0	0.5-1.0
2206BR	0.5	0.5	1.0	0.5-1.0
2207BR	0.5	1.0	1.0	0.5-1.0
2208BR	0.5	1.0	1.0	0.5-1.0
ATCC 19977	1.0	1.0	1.0	1.0

M. abscessus colonies were recovered from mice following four weeks of daily treatment with T405. The first six isolates, labeled 2201BR-2206BR, appeared smooth and isolates 2207BR and 2208BR appeared rough at the time of their isolation. ATCC 19977 is the parent isolate. Findings from three biological replicates of MIC assessments are shown in columns 2, 3 and 4. The last column shows the T405 MIC range for each isolate.

Table S2. *p*-value comparisons for lung CFU burdens between PBS and T405 or imipenem (IMI) treated mice

Timepoint	p-value (PBS vs T405)	p-value (PBS vs IMI)
**Week +2**	0.06	0.042
**Week +4**	0.006	0.007

Two-tailed *t*-tests using lung CFU data of each mouse were undertaken to determine p-values.

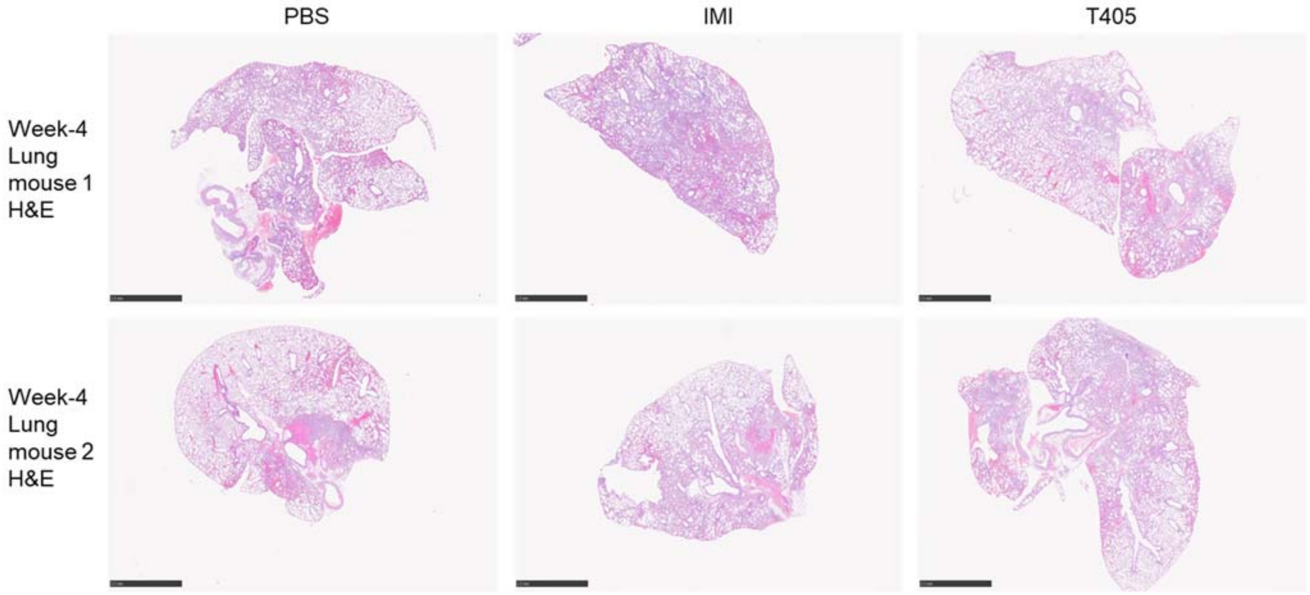

Figure S1. **Standard section of lungs of mice stained with Hematoxylin & Eosin:** C3HeB/FeJ mice were infected with *M. abscessus* and after one week treated with 1x phosphate buffered saline, pH 7.4 (PBS), or 200 mg/kg imipenem (IMI) or 300 mg/kg T405. 250 mg/kg probenecid was administered along with imipenem or T405 to reduce the rates of their clearance from kidneys. All treatments were administered twice daily, 7 days a week. Seven mice per treatment group were allocated for the four-week time point. Five mice were allocated for determination of *Mab* burden in the lungs and the remaining two mice per group were allocated for histopathological study. Lungs were harvested after four weeks of treatment, fixed in 10% buffered formalin, embedded in resin, sectioned, stained and imaged. Digital images of representative Hematoxylin and Eosin-stained sections are shown here. Scale bar = 2.5 mm.

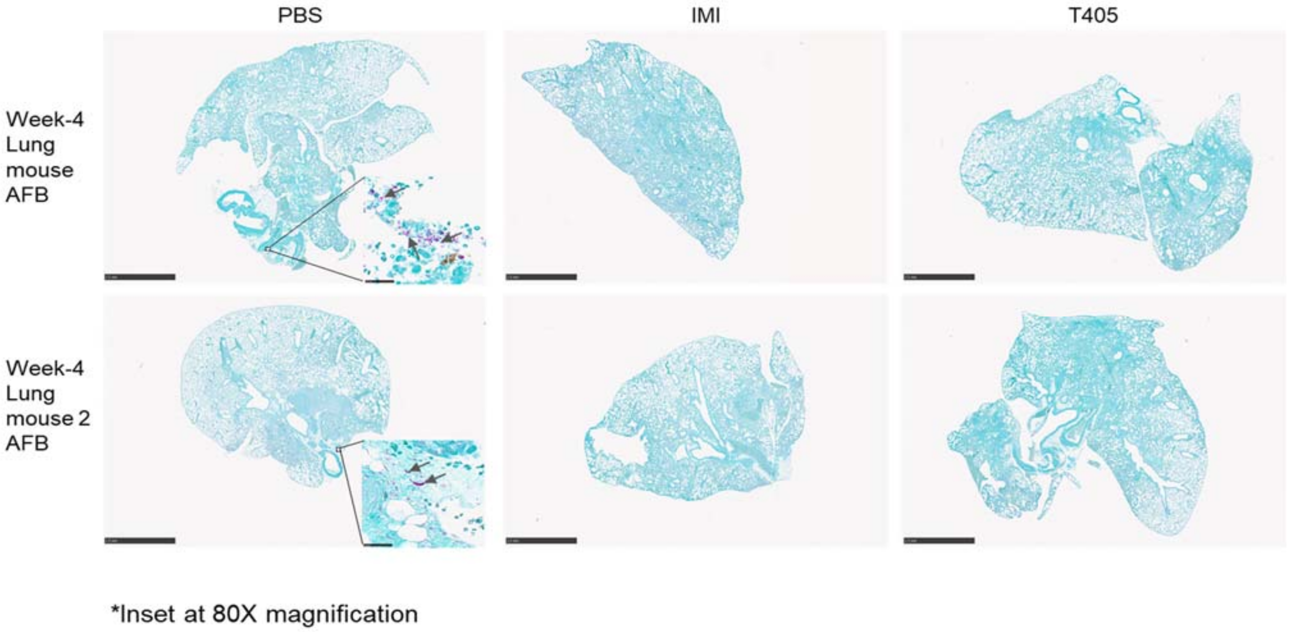

Figure S2. **Standard section of lungs of mice stained for acid-fast bacilli:** C3HeB/FeJ mice were infected with *M. abscessus* and after one week treated with 1x phosphate buffered saline, pH 7.4 (PBS), or 200 mg/kg imipenem (IMI) or 300 mg/kg T405. 250 mg/kg probenecid was administered along with imipenem or T405 to reduce the rates of their clearance from kidneys. All treatments were administered twice daily, 7 days a week. Seven mice per treatment group were allocated for the four-week time point. Five mice were allocated for determination of *Mab* burden in the lungs and the remaining two mice per group were allocated for histopathological study. Lungs were harvested after four weeks of treatment, fixed in 10% buffered formalin, embedded in resin, sectioned, stained for acid-fast bacilli (AFB) and imaged. Digital images of representative AFB-stained sections are shown here. Scale bar = 2.5 mm. Inset in PBS group shows magnification of a section of tissue with AFB staining bacilli. Scale bar for inset = 25 μm.

General Methods and Instrumentation

All reagents and starting materials were purchased and used without further purification unless otherwise indicated. Anhydrous solvents were dried using an LC Technology Solutions (Salisbury, MA) SPBT-1 solvent purification system. Silica gel chromatography was performed using Sorbtech Silica Gel (60 Å, 40-75mm particle size) or RediSep Rf disposable flash columns (60 Å, 40-63 µm irregular particle size) on a Teledyne ISCO (Lincoln, NE) CombiFlash EZ Prep. Preparative HPLC was carried out on the same instrument outfitted with a Phenomenex (Torrance, CA) Luna 10µ C18(2) 100 Å column (250 × 21.20 mm ID). All ^{1}H- and ^{13}C-NMR spectra were recorded on a Bruker (Billerica, MA) UltraShield 400 MHz Avance spectrometer. The Johns Hopkins Chemistry Department Mass Spectrometry Facility determined exact masses by high resolution ultra-performance liquid chromatography–electrospray ionization mass spectrometry (UPLC-ESIMS) using a Waters (Milford, MA) Acquity/Xevo-G2.

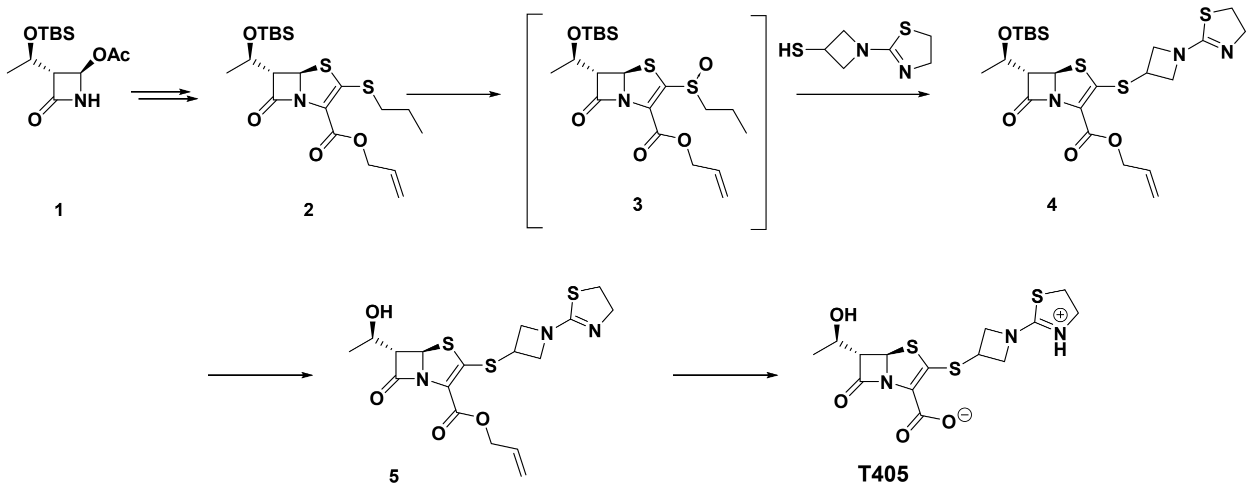

Supplemental Scheme 1. Large-scale synthesis of **T405**

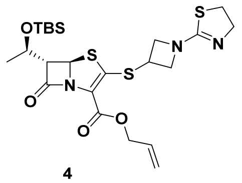

4

Allyl (5R,6S)-6-((R)-1-((tert-butyldimethylsilyl)oxy)ethyl)-3-((1-(4,5-dihydrothiazol-2-yl)azetidin-3-yl)thio)-7-oxo-4-thia-1-azabicyclo[3.2.0]hept-2-ene-2-carboxylate (4).

Compound **2** was synthesized as previously reported from **1**. (https://doi.org/10.1038/s42003-020-01475-2) In a 250 mL round bottom flask **2** (10 g, 23 mmol) was dissolved in hexafluoro isopropanol (54 mL). Urea peroxide (2.6 g, 27 mmol) was then added to the solution portion-wise and stirred for 14 h. Upon completion of the reaction, the solvent was removed *in vacuo* to give a yellow oil. The oil was then redissolved in ethyl acetate (50 mL) and passed through a pad of silica

gel. The silica gel was then washed with ethyl acetate (3× 100 mL). The resulting filtrate was concentrated *in vacuo* to give crude intermediate **3** as an oil, which was dissolved in acetonitrile (158 mL) and cooled to 0 ˚C under a nitrogen atmosphere. The side chain 1-(4,5-dihydrothiazol-2-yl)azetidine-3-thiol hydrochloride (4.8 g, 23 mmol), was charged in a separate flask and suspended in acetonitrile (67 mL). *N,N*-Diisopropylethylamine (12.6 mL, 72 mmol) was then added dropwise to the sidechain mixture and cooled to 0 ˚C. The basified sidechain mixture was then added to the flask containing intermediate **3** in one portion. The reaction proceeded for 3 h before being quenched with saturated brine solution (50 mL). The organics were extracted from the mixture with ethyl acetate (3× 150 mL). The organic extracts were combined, dried with anhydrous sodium sulfate, filtered, and concentrated *in vacuo*. The resulting oil was purified using silica-gel flash chromatography with a gradient of hexane to ethyl acetate. The product was then triturated using diethyl ether to yield an amorphous, white powder (8.2 g, 66%). **4**: ^{1}H NMR (400MHz, $CDCl_3$) δ = 5.94 (tdd, *J* = 5.6, 10.5, 17.2 Hz, 1H), 5.66 (d, *J* = 1.2 Hz, 1H), 5.41 (dd, *J* = 17.2, 1.5 Hz, 1H), 5.25 (dd, *J* = 10.4, 1.3 Hz, 1H), 4.71 (dtABq, *J* = 1.4, 5.4, 13.4 Hz, 2H), 4.50 (sym m, 1H), 4.32 (sym m, 1H), 4.25 (sym m, 1H), 4.18 (sym m, 1H), 4.04 (t, 7.4 Hz, 4H), 3.71 (dd, *J* = 1.4, 4.6 Hz, 1H), 3.40 (dt, *J* = 1.7, 7.4 Hz, 2H), 1.26 (d, *J* = 6.3 Hz, 3H), 0.89 (s, 9H), 0.08 (s, 6H). ^{13}C NMR (101MHz, $CDCl_3$) δ = 172.2, 159.8, 131.8, 118.5, 118.2, 77.4, 71.9, 65.8, 65.3, 64.6, 59.7, 59.2, 36.5, 36.0, 25.8, 22.6, 18.1, -4.2, -5.0. HRMS (UPLC), $C_{23}H_{35}N_3O_4S_3Si$ [M+H$^+$] calculated: 542.1632; found: 542.1647.

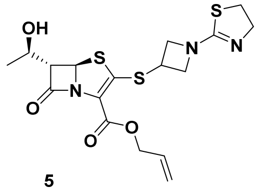

5

Allyl (5R,6S)-3-((1-(4,5-dihydrothiazol-2-yl)azetidin-3-yl)thio)-6-((R)-1-hydroxyethyl)-7-oxo-4-thia-1-azabicyclo[3.2.0]hept-2-ene-2-carboxylate (5).

Compound **4** (8.2 g, 15 mmol) was charged in a 250 mL round bottom flask and dissolved in tetrahydrofuran (THF) (43 mL) with stirring. To the solution acetic acid (16.4 mL, 290 mmol) was added followed by tetra-*n*-butylammonium fluoride 1M in THF (30 mL, 30 mmol). The reaction mixture was stirred for 14 h before being quenched with saturated aqueous bicarbonate solution for 10 min. The organics of the mixture were extracted into ethyl acetate (3× 150 mL). The organic layer was washed with brine, dried with anhydrous sodium sulfate, filtered, and concentrated *in vacuo*. The resulting oil was purified with flash silica-gel chromatography with a gradient from hexanes to ethyl acetate. The product was further triturated with diethyl ether to give an amorphous, white powder (5.2 g, 80%). Characterization data matched the literature (1).

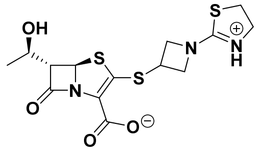

T405

(5R,6S)-3-((1-(4,5-dihydrothiazol-2-yl)azetidin-3-yl)thio)-6-((R)-1-hydroxyethyl)-7-oxo-4-thia-1-azabicyclo[3.2.0]hept-2-ene-2-carboxylic acid (T405).

Compound **5** (5.3 g, 12 mmol), triphenyl phosphine (293 mg, 1.1 mmol), and sodium benzenesulfinate (2.7 g, 12 mmol) were charged in a flask and suspended in a 2:1 ethyl acetate to water mixture (186 mL). Nitrogen gas was then bubbled through the mixture for 15 min to purge oxygen. While stirring under a nitrogen atmosphere, palladium tetrakis(triphenylphosphine) (43 mg, 0.03 mmol) was added to the mixture and let stir for 2 h. Upon completion of the reaction, the aqueous and organic layers were separated, and the organic layer was washed twice with water. The aqueous layers were combined and partially concentrated *in vacuo* before dilution with methanol (16 mL). Macroporous polystyrene-2,4,6- trimercaptotriazine (MP-TMT) resin (1.2 g, Matrix Innovation, Quebec City, Canada) was then incubated with the mixture overnight at 0 ˚C with agitation. The MP-TMT resin was then filtered from the solution and washed twice with methanol (2× 5 mL). The filtrate was partially concentrated *in vacuo* and the resulting mixture was purified using a column (7 cm × 30 cm) of HP-20 resin. A gradient of 0% to 5% ethanol in water was used to elute **T405**. The fractions were then partially concentrated *in vacuo* and further lyophilized to yield an off-white powder (2.75g, 58%). Characterization data matched the literature (1).

^{1}H NMR of Compound **4**

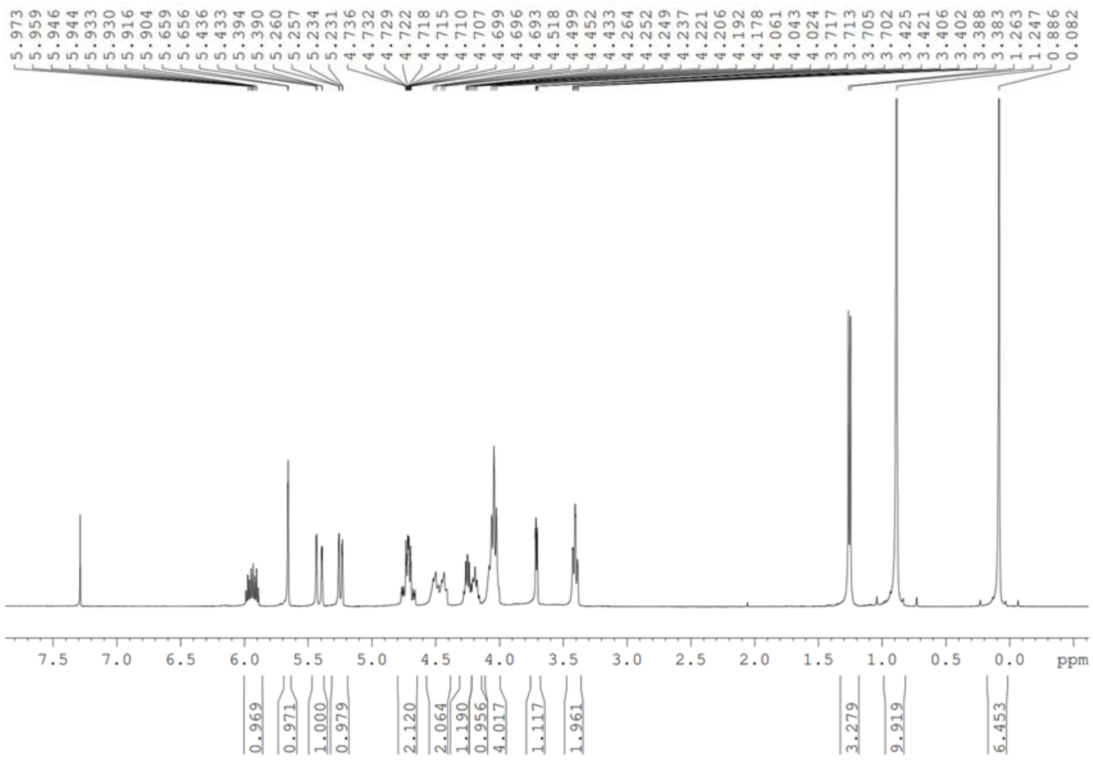

^{13}C NMR of Compound **4**

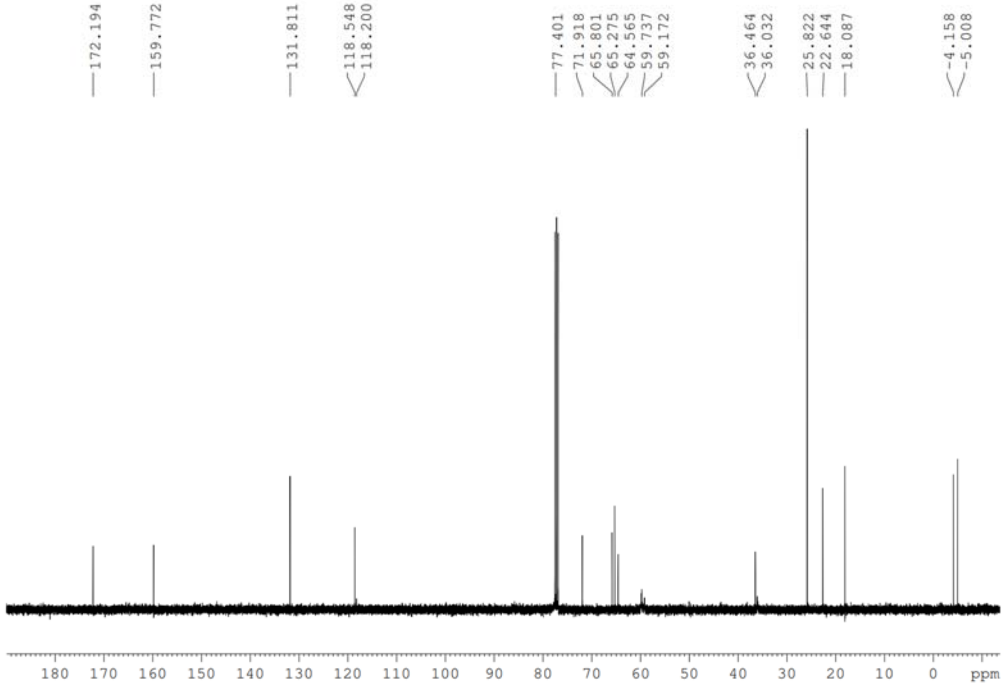

REFERENCE

1. Batchelder HR, Story-Roller E, Lloyd EP, Kaushik A, Bigelow KM, Maggioncalda EC, Nuermberger EL, Lamichhane G, Townsend CA. 2020. Development of a penem antibiotic against Mycobacteroides abscessus. Commun Biol 3:741.
